# Graph convolution network for fraud detection in bitcoin transactions

**DOI:** 10.1038/s41598-025-95672-w

**Published:** 2025-04-01

**Authors:** Ahmad Asiri, K. Somasundaram

**Affiliations:** 1 Department of Mathematics, Applied College at Mahail Aseer, King Khalid University, Abha, Saudi Arabia; 2 Department of Mathematics, Amrita School of Physical Sciences - Coimbatore, Amrita Vishwa Vidyapeetham, Coimbatore, India

**Keywords:** BITCOIN, Cryptocurrency, Machine learning, Deep learning, Graph convolution network, Mathematics and computing, Computer science

## Abstract

Anti-money laundering has been an issue in our society from the beginning of time. It simply refers to certain regulations and laws set by the government to uncover illegal money, which is passed as legal income. Now, with the emergence of cryptocurrency, it ensures pseudonymity for users. Cryptocurrency is a type of currency that is not authorized by the government and does not exist physically but only on paper. This provides a better platform for criminals for their illicit transactions. New algorithms have been proposed to detect illicit transactions. Machine learning and deep learning algorithms give us hope in identifying these anomalies in transactions. We have selected the Elliptic Bitcoin Dataset. This data set is a graph data set generated from an anonymous blockchain. Each transaction is mapped to real entities with two categories: licit and illicit. Some of them are not labeled. We have run different algorithms for predicting illicit transactions like Logistic Regression, Long Short Term Memory, Support Vector Machine, Random Forest, and a variation of Graph Neural Networks, which is called Graph Convolution Network (GCN). GCN is of special interest in our case. Different evaluation parameters such as accuracy, ROC and F1 score are analyzed for different models. Our experimental results show that the proposed GCN model gives the accuracy $98.5\%$, the AUC 0.9444 and the RMSE 0.1123, which concludes that our GCN is better than the existing models, in particular with the model proposed in Weber et al. (Anti-money laundering in bitcoin: experimenting with graph convolutional networks for financial forensics, 2019. http://arxiv.org/abs/1908.02591).

## Introduction

Cryptocurrency has been used these days more frequently than usual. It is a digital currency, that is, the encrypted data strings which only exist electronically. They are not decentralized; in other words, they are not issued by the government. Cryptography algorithms are used to create cryptocurrency and they are validated through mining^1^. If the miners are successful, they will receive cryptocurrency as a reward^2–4^. These electronic currencies are stored in a structure and it is called blockchain. Blockchain gives information with node details in a distributed environment. The information cannot be edited or ruined. Transactions occur in blocks of data, and each block has a pointer to the previous block. The arrival of Bitcoin has triggered the popularity of cryptocurrency^5^. However, this recurring use of cryptocurrency has captured the attention of many scammers. Cryptocurrency scams occur through many means; ransomware attack, financial crimes, Ponzi schemes, etc. However, cryptocurrency is a secure network that provides anonymity to its users. Scammers take advantage of this and use this facility to camouflage themselves.

### Bitcoin

Bitcoin is one of the first cryptocurrency. It works by using blockchain and is more valuable than others. The bitcoin blockchain can be viewed as a shared ledger in which every transaction is recorded and cannot be changed. Each block has a unique hash pointer which points to the previous block. These points are the links between each block. Nodes in the network would validate the new transaction and is added to the ledger or the blockchain which is then published to the rest of the nodes. When mining is done to create new bitcoin, the miner gets the unique hash value of the new coin using algorithms. In recent days, the Bitcoin’s pseudonymity is used for illegal activities such as goods transport and banking transactions^6^.

### Anti money laundering in cryptocurrency

Weaker sections of society struggle to be included in the financial process of the country. Financial inclusion basically means providing access to these groups of society to participate in the country’s financial development by providing them with financial services and products at a reasonable cost. But these marginal groups are restricted access to these systems, which is an accidental repercussion of Anti Money Laundering (AML) regulations. Unfortunately, these regulations cannot be avoided to maintain the protection of our economy. AML also results in relatively higher costs on products. AML regulations are mandatory and must be followed because many illegal transactions are identified and thus avoided.

### Literature review

The financial marketing studies say that Bitcoin purchase increases in 2024. While the volume of transactions increases, fraudulent activities also increased^7^. Dhanya^8^ collected the Bitcoin data from 2013 to 2018 and proposed rollover techniques for better prediction of accuracy in the time series model. She considered the Bitcoin date from The bitcoin data from 2013 to 2018. Also recent machine learning methods overcome the time series mode. In this paper, we consider the data till 2024. The proposed security protocol for Bitcoin is one of the important aspects. Recently, Badertscher et al.^9^ proposed a security proof for the Bitcoin protocol. They proposed a secure protocol for global clock and hash functions. As an application and case study, Kumar et al.^10^ proposed a model for blockchain-based land registration. In an unpublished Arxiv paper, Weber et al.^6^ proposed different machine learning models to predict illicit transactions. They have found precision, recall, F1 score, and micro-average. For illicit transactions with GCN, their precision value is 0812, Recall score is 0.512 and R1 score is 0.628. Our experimental results are shown in the section 4.1, which improved their results. Chen et al.^11^ gave a detailed survey on different machine learning methods to detect suspicious transactions on Bitcoin^11^. In this paper, they have an analysis of some of the important aspects such as risk scoring, link analysis and behavioral modeling. Klaus Grobys et al.^12^ proposed a statistical distribution model for stolen Bitcoins. Features extractions are one of the important in the study of Bitcoin transactions. Vlahavas et al.^13^ used a clustering algorithm for feature extractions. Pranav Nerurka^14^ proposed a graph attention network (GAT2) with accuracy of $92\%$ to identify illegal transactions.

### Motivations and key contributions

Identifying and preventing the illegal transactions during bitcoin transactions are challenging problems in the private financial market. The actor called a “miner” (person/machine) will participate and validate all the Bitcoin transactions. The miner is considered the most important person for the creation of new cryptocurrencies and their validations. The minor’s decision is based on his mathematical model used for the transactions. Even a small error in the prediction model leads to a huge financial loss for the transacting people. For example, during the 10-year period 2011–2021, nearly 1.7 million Bitcoin units were stolen due to criminal activity, with losses exceeding 700 million^12^. So, $100\%$ accuracy will be the goal of any miner. The present methods give a maximum accuracy of $97\%.$ This $3\%$ gab needs to addressed. In this paper, we proposed a graph-based deep learning algorithm to find these illegal transactions that gives the precision of $98.5\%.$

**Fig. 1:**
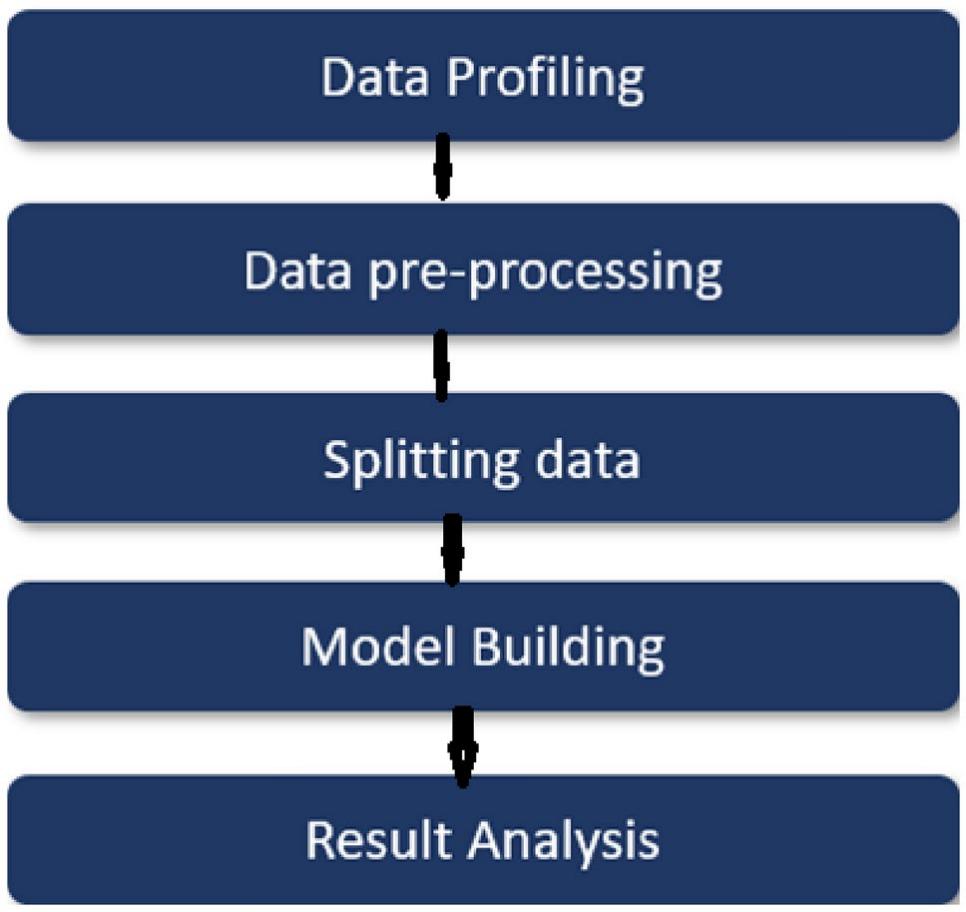
Proposed methodology.

Our methodology is shown in Fig. 1.

*Key contributions*:

- A graph based evaluation method is proposed for a fraud detections during Bitcoin transactions.
- We proposed a GCN model for the classification and early detection of illicit transactions.
- Different evaluation parameters such as accuracy, ROC and F1 score are analysed for the better performance.
- Our experimental results show that the proposed GCN model gives $98.5\%$ accuracy, which is better then the existing models.

## Data preprocessing

Elliptic^15–17^ is a company that provides a platform for cryptocurrency exchanges and financial services with Anti-Money Laundering software. In this paper, we use the Elliptic Bitcoin Dataset, which maps the Bitcoin transactions to the licit category versus the illicit category.

### Construction of graph

A transaction graph is a directed graph, each transaction is considered as vertices (nodes) and the transaction flow from one to another is represented as an edge (link). We have considered 166 features correlate with each node, and each node is labeled as licit, illicit, or unknown. The categories such as miners, wallet providers, and exchanges are considered as licit and the categories such as ransomware, Ponzi schemes, and malware are marked as illicit. Our graph consist of 203769 nodes and 234,355 edges. Of the 27769 nodes, there are 4,545 nodes labeled as (illicit) class-1 and 42,019 nodes are labeled as (licit) class-2. All other nodes are classified as unknown nodes (class-3). The details are shown in Table 1.

**Table 1:** Data set.

# Nodes (transactions)	203,769
# Edges (money flow)	234,355
# Time steps	49
# Illicit (class-1)	4,545
# Licit (class-2)	42,019
# Unknown (class-3)	157,205

#### Graph centrality measures

**Fig. 2:**
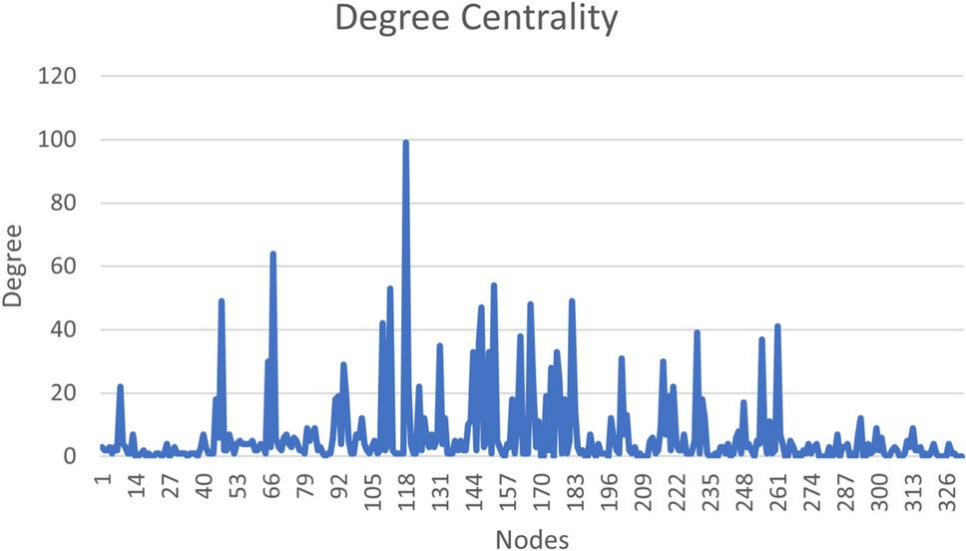
Degree centrality.

Graph centralities are important graph parameters to identify the most significant nodes. Various centrality measures are in the literature. In this work, we use the important centralities, namely, degree and closeness centralities. Degree Centrality of a node in a graph is the average degree of the node. This centrality shows the number of neighboring nodes for a given node. The degree centrality of our data is shown in Fig. 2.

**Fig. 3:**
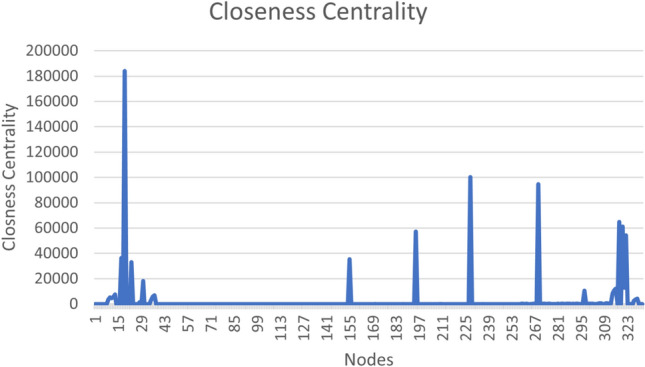
Closeness centrality.

Closeness centrality is another measure that gives a node more connectivity in terms of information dissemination to other nodes. The average fairness can be obtained from this centrality. It is obtained by taking the average of the shortest path lengths from the node to every node in the network. The closeness centrality of our data is shown in Fig. 3.

**Fig. 4:**
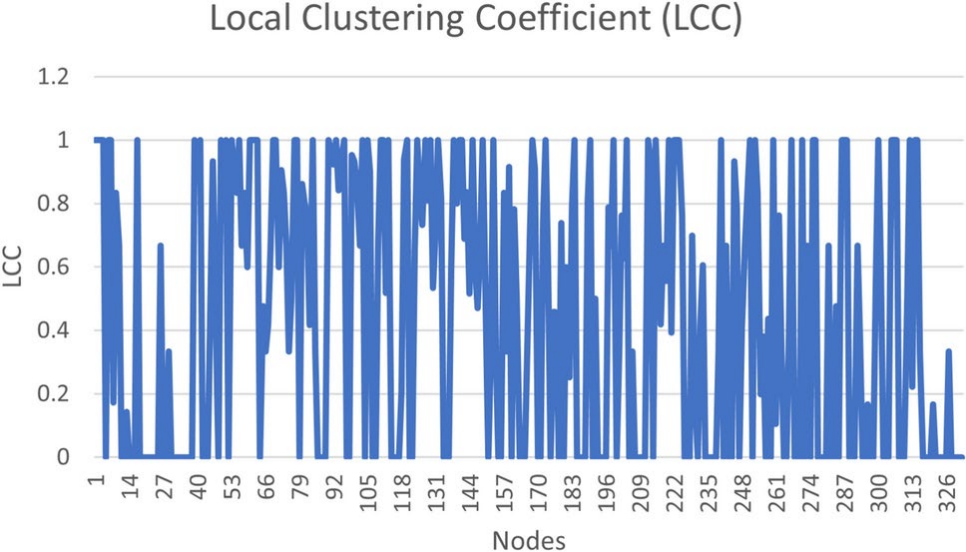
Local clustering coefficient.

Cluster centrality is one of the important graph centrality measures revealing the most important data clusters. The Local Clustering Coefficient (LCC) of a node *u* is a ratio between the number of links with in the neighbours of *u* and total number of links in the graph. We show the LCC for our data in Fig. 4.

### Exploratory data analysis

**Fig. 5:**
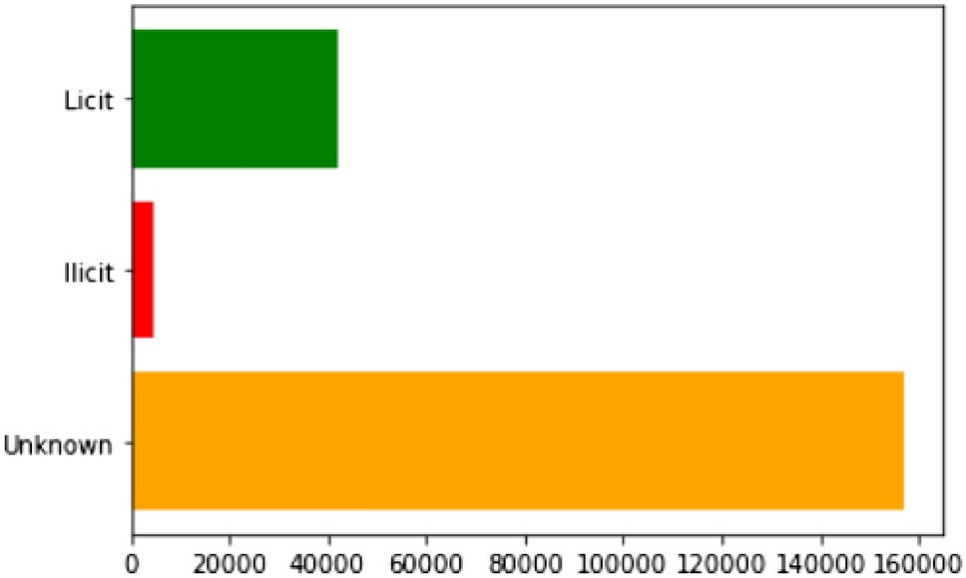
Bar graph of the count of three classes.

**Fig. 6:**
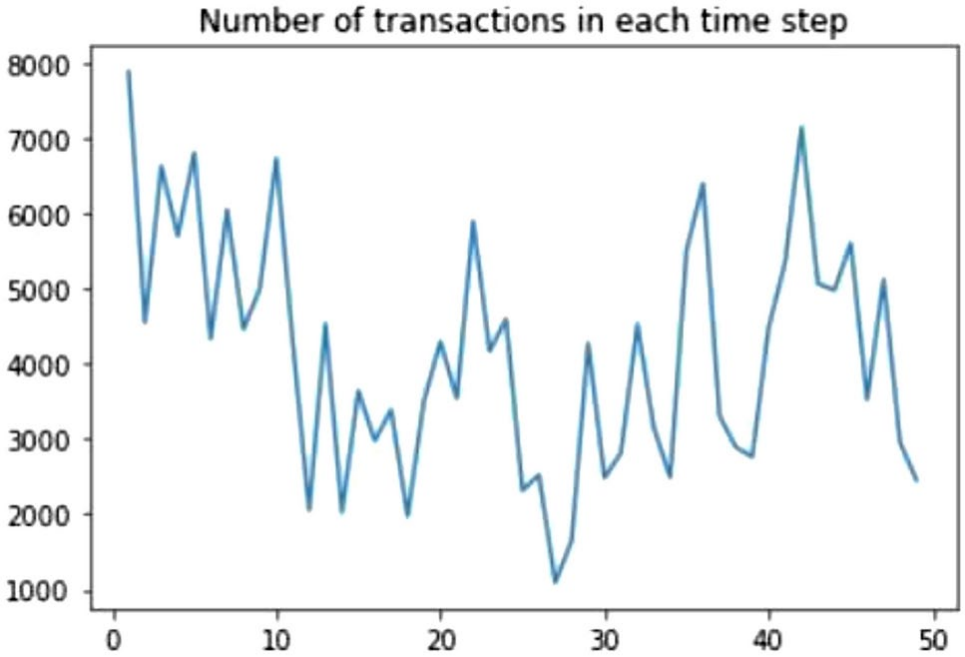
Number of Transactions in each time step.

**Fig. 7:**
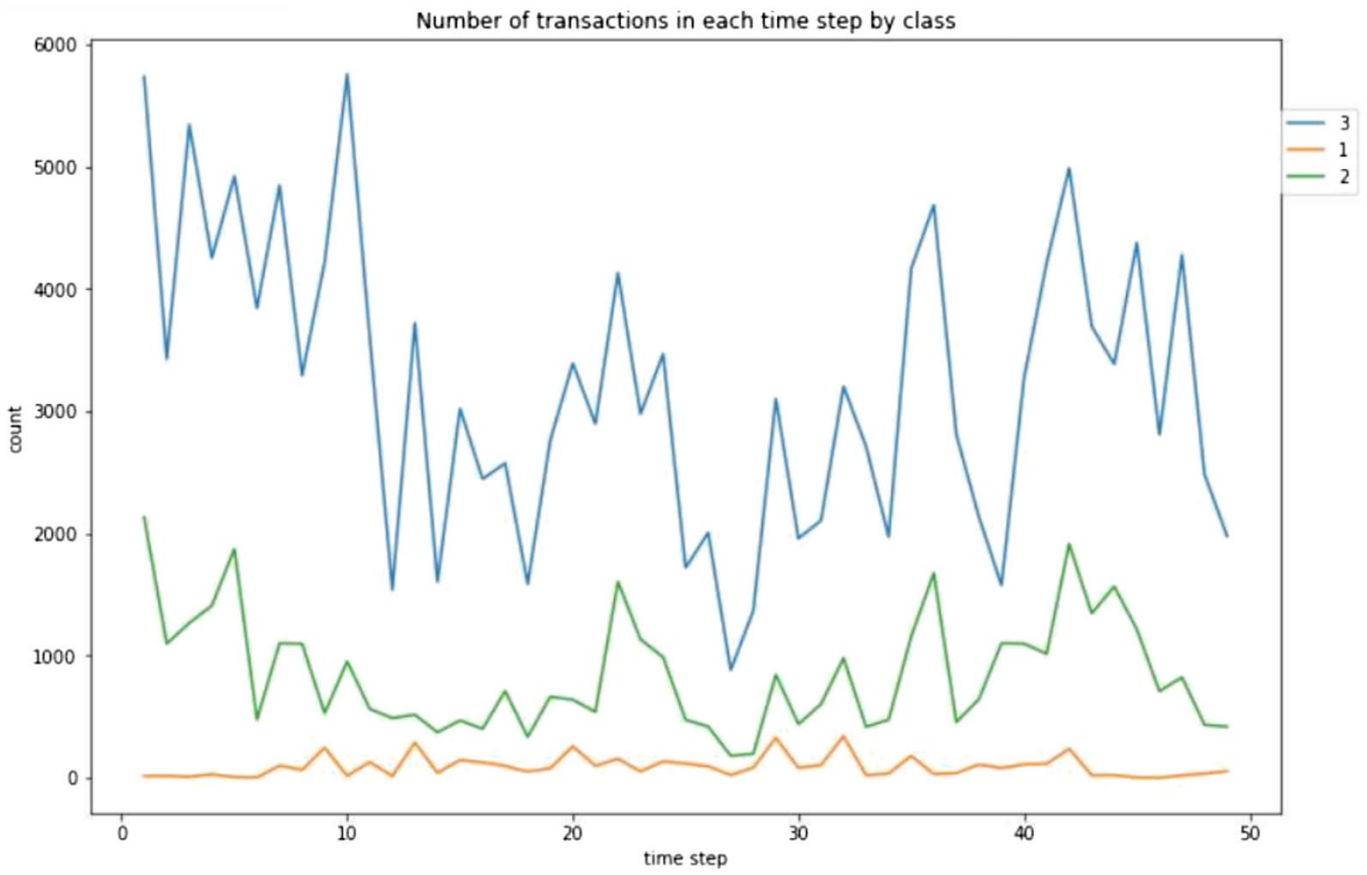
Number of Transactions in each time step.

Data preprocessing is a step in which the data is taken and cleaned so that it is easier to understand and work with. We have taken the entire data set and filled in the missing values. We use the mean value of the data to fill in the missing values in the data set. We normalize the data set with nearest neighbor algorithm. Our data have three different files in our dataset; features, edges, and classes. We have merged features and classes while implementing each algorithm. Exploratory data analysis is performed to visualize trends and patterns in the data. We have done several visualizations which include:

- Bar graph of the count of three classes, which is shown in Fig. 5.
- Line graph of the number of transactions in each time step, which is shown in Fig. 6.
- Line graph of the number of transactions for each class in each time step, which is shown in Fig. 7.

We have given the plots of each of them, respectively.

#### Features

Feature selection is one of the main aspects of data pre-processing. We combine the three graph centrality measures and the correlation coefficient for the feature selection. There are 166 attributes linked with each node. First 94 of these 166 features constitute local information regarding the transaction. For example,

- Transaction charges.
- Time step
- $\#$ inputs or outputs
- Output values such as the average bitcoin received and the average number of incoming transactions.

**Fig. 8:**
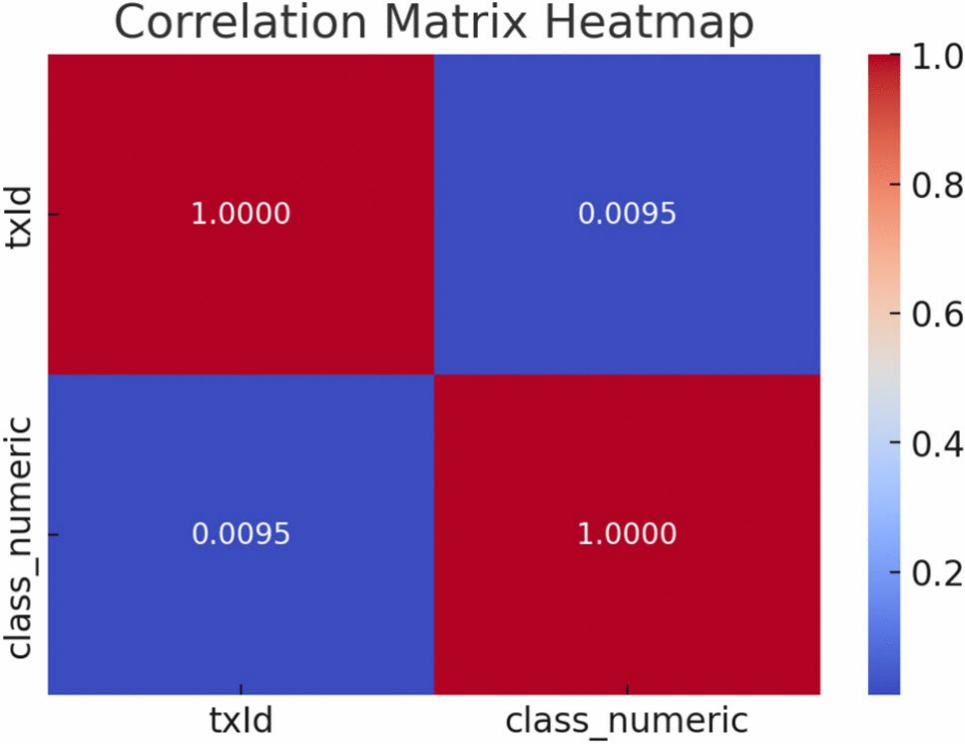
Heatmap of the correlation matrix.

There are 72 other attributes are labeled as aggregated features; there features are obtained by one step forward or backward transactions. These features take the statistical parameters such as standard deviation and correlation coefficients^6^. The heat map of the correlation matrix is shown in Fig. 8. The color gradient represents the strength of the correlation. Red indicates a strong positive correlation. Blue indicates a strong negative correlation. Values near 0 indicate weak or no correlation.

#### Temporal information

Temporal information of a data gives information about time steps and different points in time. We obtained the temporal information from the Elliptic dataset. First, we merge the data with timestamp and transaction ids. The timestamp is the record of when a transaction has occurred in the bitcoin network. That is, it records the date and time. For example, the txId 230425980 had the timestamp 2016-12-01 10:15:30 and the txID 5530458 had the timestamp 2016-12-01 10:17:45. We have 49 different timestamps in our data set that are evenly spaced. In every time step in the blockchain, the transactions are computed every three hours. For the different time step transactions, there are no edges connecting them. Figures 6 and 7 show the number of transactions in each time step.

## Methodology

Our major aim is to find the anomalies, that is, the granules that are the illegal transactions in the vast sea of transactions that we have, which will lead to letting guiltless civilians continue their transactions uninterrupted and capturing the frauds. When talking algorithm-wise, we have to decrease false positive rates while keeping false negative rates as such. Random Forest classifier and Logistic Regression algorithm are best used to do this particular task. We are also using Graph Convolution Networks algorithm for this too. Additionally, we are introducing the Long Short Term Memory algorithm and the Support Vector Machine for this task for our dataset. We have to classify the unlabeled nodes into either the licit category or the illicit category. We consider the learning rate to be one of the hyperparameters for our models. There are several techniques for hyperparameter tuning. We considered different techniques for parameter tuning. For the invalid data, we ensure that the hyperparameter tuning is performed together with cross-validation.

### Logistic regression

Machine Learning (ML) algorithms are powerful algorithms for data classification and prediction. Logistic regression (LR) is one of the supervised ML algorithms for binary classifications. This is a statistically based algorithm^18–21^. As a result, the output of the categorical dependent variable is predicted. There will also be a set of independent variables. This algorithm fits a curve of the ’S’ shape called the Sigmoid curve. The signature or logistic function maps any real values to probabilities. We get the equation of Logistic Regression from the equation of the straight line.

$$\begin{aligned} log{\left[ \frac{y}{1-y}\right] }=b_0+b_1x_1+b_2x_2+\cdots +b_nx_n. \end{aligned}$$

We apply the GridSearchCV technique for the hyperparameter tuning which improves accuracy. The accuracy value is $97.52\%$.

### Random forest classifier

Random Forest algorithm is also a supervised ML algorithm with ensemble learning. We make use of many classifiers instead of just one. That is, we will use many decision trees and take the output, which will be maximum in number. This is ensemble learning. This will ensure better performance of the model with the following steps:

- We build trees linked to the selected data points in the training set.
- Pick the number of decision trees.
- We then vote with majority for the output of the decision trees, and the category that wins would be the final output.

We use Max_features for selection of different features in the trees. Max_depth is used to fix the number of splits in each tree. Each tree will take a certain number of data, which is decided by the max_sample function. Similarly to logistic regression, we use the GridSearchDV technique for hyperparameter tuning.

### Support vector machine

Another important supervised ML algorithm is the Support Vector Machine (SVM) algorithm. By creating a line, or rather a decision boundary, the algorithm classifies the data into different classes. The hyperplane is the best decision boundary. This SVM was developed by Vapnik et al.^22^. This SVM will work for both linear and non-linear models^23^.

### Long short term memory networks

**Fig. 9:**
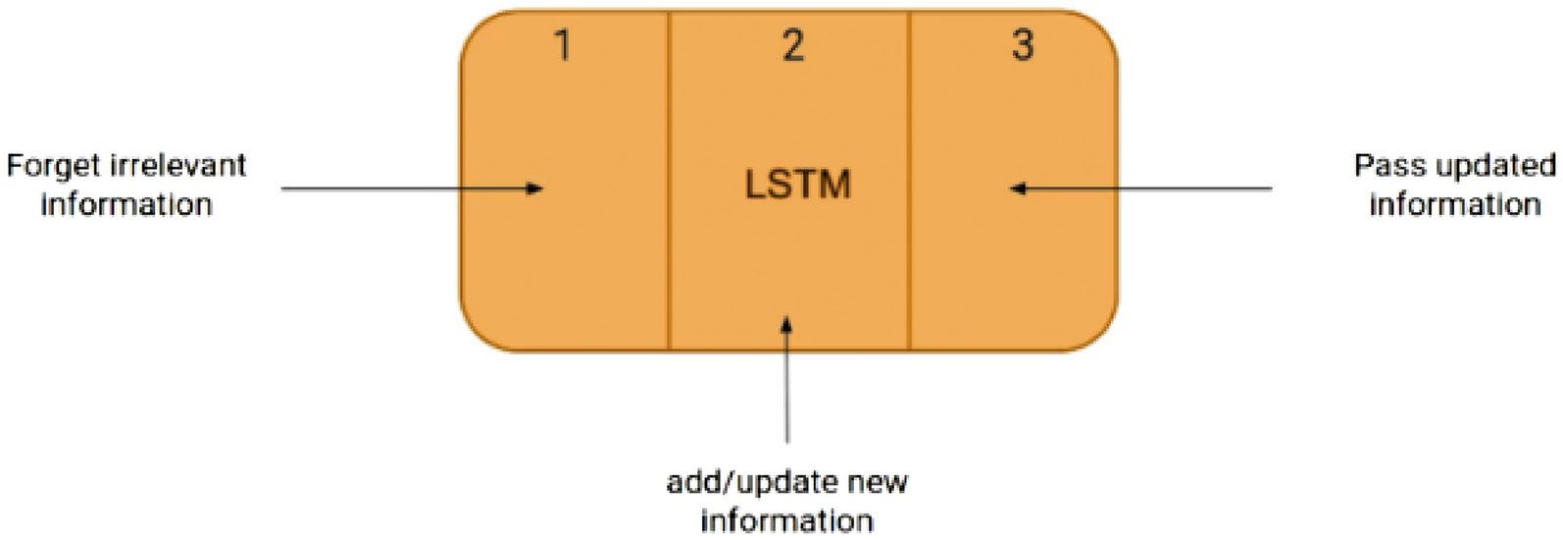
Architecture of LSTM.

Recurrent Neural Network is a variation of the neural network. Long Short Term Memory Network (LSTM) is an improved version of RNN. This will allow us to store the transition information. There are four interactive layers in LSTM networks. The architecture is shown in Fig. 9.

The first portion known as the Forget gate decides on remembrance of the previous transactions or ignores the redundant information on transactions. The cell will learn the new transaction information and take this information to the next phase. The updated transaction information will be passed to subsequent transactions and the cell update this process.

### Graph convolutional networks

In recent years, graphs networks have been used extensively because of their flexibility. Graphs naturally arise in many real-world applications, including social analysis^24^, fraud detection^25,26^, traffic prediction^27^, computer vision^28^, and many more. Graph based Deep learning models are powerful. Graph structured data are used in graph convolutional models^6,29–32^. They are used for prediction, labeling, link prediction, etc. Convolutional Neural Networks (CNN) can process more complex data. We have taken an algorithm which combines both graphs and CNN called Graph Convolutional Networks (GCN) which can work directly on graphs. Chanchal et al.^33^ and Aarthi et al.^34^ gave two nice survey papers on Image Data, the authors extensively used the neural network algorithms for the classifications. Fruchterman-Reingold layout is another form of graph visulation for better layout. In Fig. we show the for the Bitcoin transaction graph for sample of 100 nodes.

**Fig. 10:**
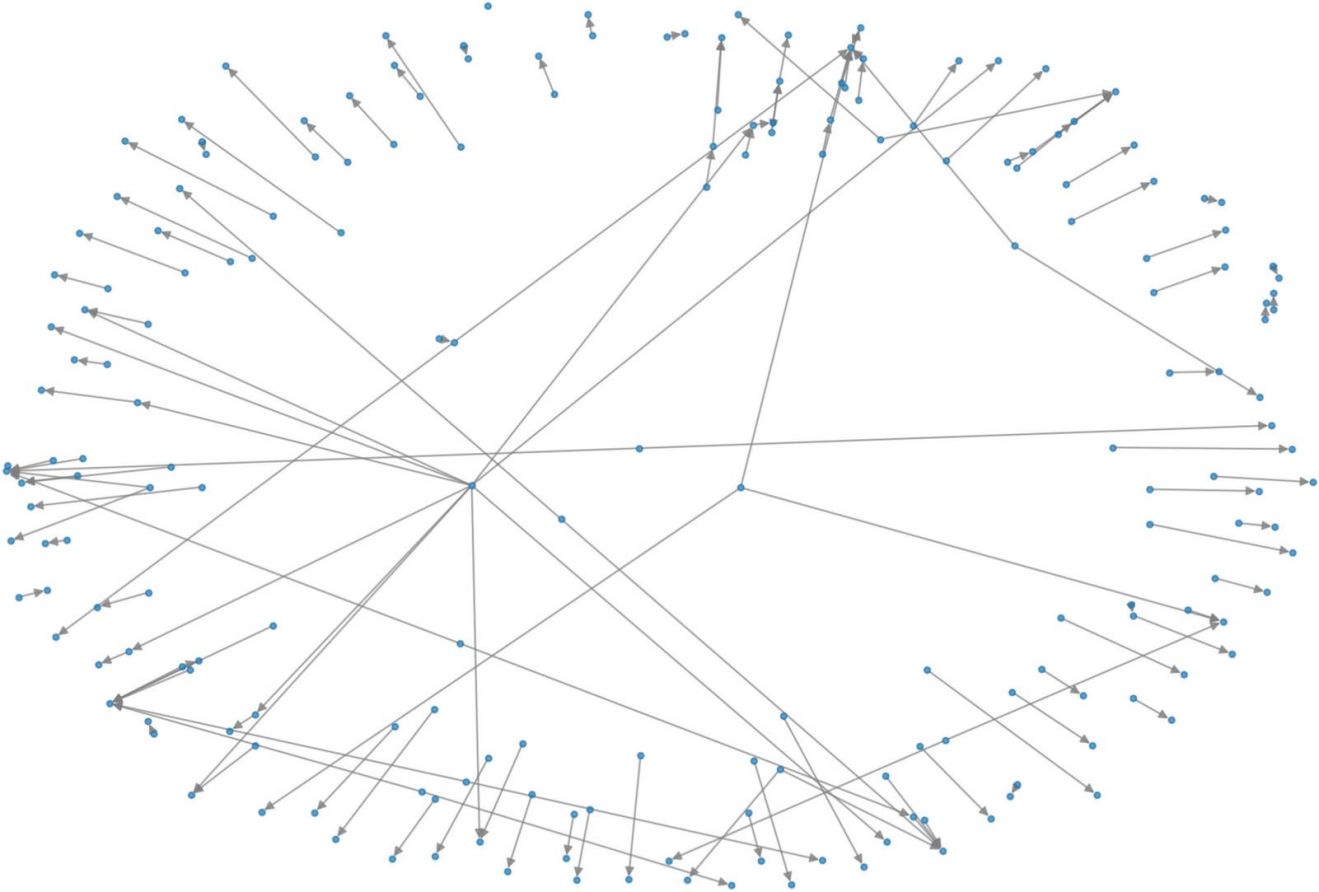
Subgraph of the data set.

**Fig. 11:**
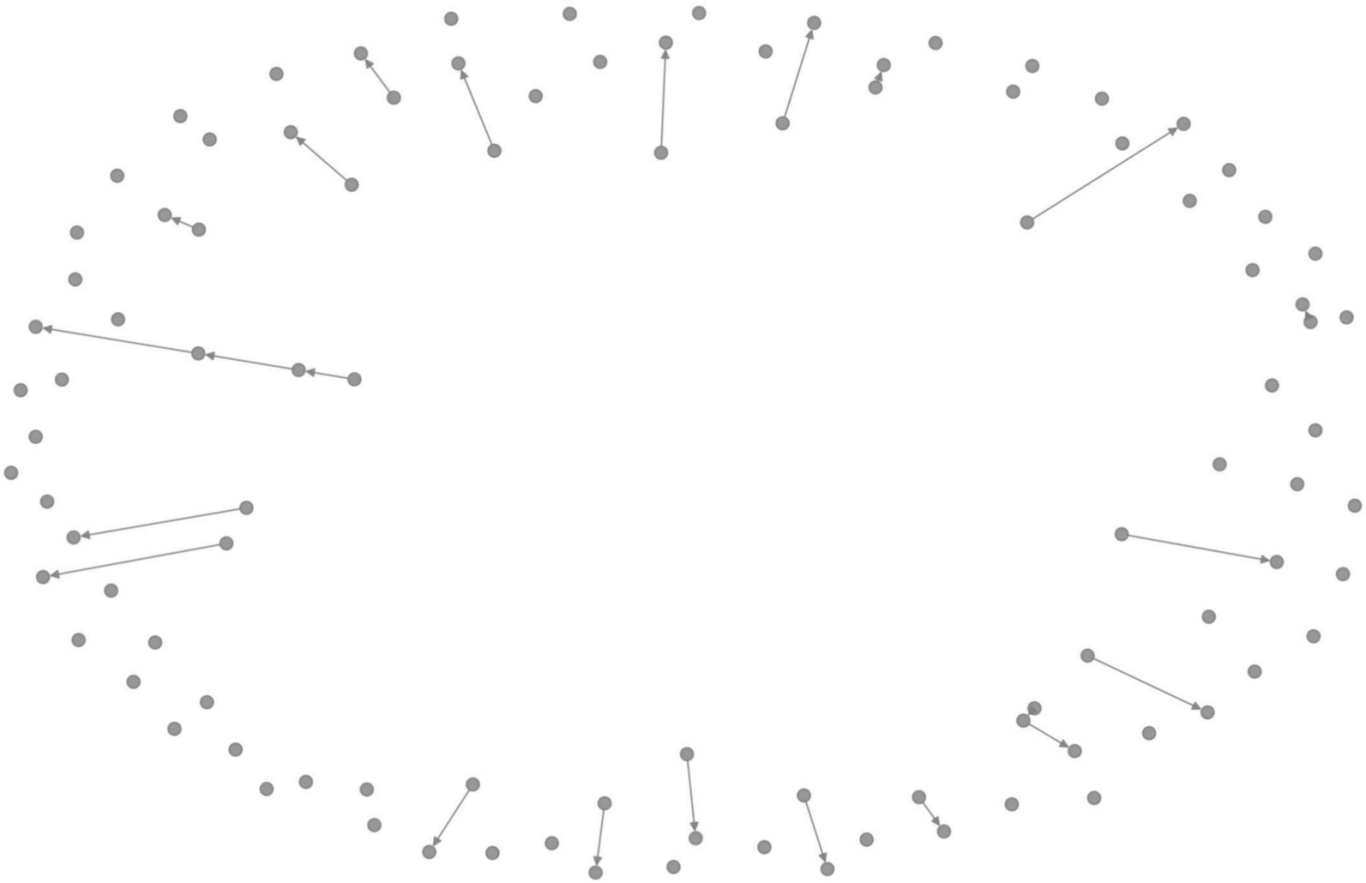
Fruchterman–Reingold layout for the Bitcoin transaction graph.

We consider a graph $G=(N, E)$ that is generated from the whole bitcoin transaction graph, here *N* represents the transactions, and *E* represents the bitcoin movement. The graph network is constructed from 234,355 edges. First we extract the graph from its edge list and then we associate its node features for training purpose. Random feature vectors are assigned to generate synthetic features. We use binary lables for classifications. We apply the attention mechanisms by adding more layers to the feature selection. To Create a Feature Matrix, we consider that each row corresponds to a node and each column corresponds to a feature. Figure 10 shows a subgraph corresponding to the Bitcoin data set that is generated from 200 nodes and their transaction, and Fig. 11 shows the Fruchterman-Reingold layout for the Bitcoin transaction graph.

**Table 2:** GNN: Accuracy for different Epoch.

Epoch	Accuracy	Loss	Validation accuracy	Validation loss
1/5	0.1432	3.2758	0.1362	1.7915
2/5	0.82473	0.54122	0.1461	1.8376
3/5	0.94578	0.18972	0.13125	1.8974
4/5	0.98021	0.08273	0.15461	1.92635
5/5	0.9856	0.05921	0.16843	1.93572

We generate the adjacency *A* and the node embedding $H^{(l)}$ matrices corresponding to the graph *G*. We use *A* and $H^{(l)}$ for our GCN at $l^{th}$ layer as input and updates the node using a weight matrix $W^{(l)}$ and gives $H^{(l+1)}$ as output. On a mathematical level. Hence,

$$\begin{aligned} H^{(l+1)} = \sigma (A'H^{(l)}W^{(l)})). \end{aligned}$$

**Fig. 12:**
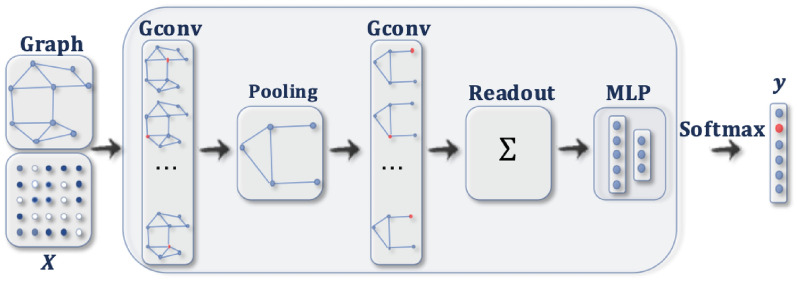
GCN architecture^35^.

$\sigma$ is the activation function for all the layers that we have used, ReLU but the output layer. For the output layer, we have used Softmax as the activation function. The embedding matrix used first is originally from the node features. We use a basic GCN model. A GCN architecture (Fig. 12) was proposed by Defferrard et al.^35^ and was used by Zonghan et al.^36^. In this paper, we use this architecture for our date set. Our GCN has different levels of mechanisms. The node tasks are carried out at end-to-end level. Using semi-supervised learning, we train our GCN model. We apply t-Distributed Stochastic Neighbor Embedding (t-SNE) for the reduction in dimensionality and use RelU as an activation function in our GCN. Our training shows better output for the next level. The values of the accuracy and loss functions for different epochs are shown in Table 2.

#### Evaluation metrics

There are several evaluation metrics for the evaluation of the DL models. In this paper we use Precision, Recall and F1 score for our model and comparison of each of these measures used in different DL models.

$Precision = \frac{{TP}}{{TP + FP}},$ where TP (True Positives) refers to the number of correctly predicted positive instances of skin cancer by a classification model. In this scenario, the classification model is trained to identify whether a given skin lesion or image is indicative of skin cancer (positive class) or not (negative class). When the model correctly predicts a positive instance, it is counted as a True Positive. FP (False Positives) refers to the number of incorrectly predicted positive cases of skin cancer by a classification model. In this scenario, the classification model is trained to identify whether a given skin lesion or image is indicative of skin cancer (positive class) or not (negative class). When the model correctly predicts a negative instance, it is counted as a False Positive.

Figure 17 shows the precision values of the comparative models and the proposed GCN model after training for 20 epochs. The findings clearly highlight the superiority of the improved GCN model over the other models, as it achieves the highest accuracy of 98.56%.

$$Recall = \frac{{TP}}{{TP + FN}}.$$

The recall measure is depicted, showing a strong correlation with the precision values obtained after training for 20 epochs. The proposed GCN model stands out with the highest recall of 0.9381, better than other DL models, shown in Fig. 18.

$$F1 Score = \frac{{2 \times Precision \times Recall}}{{Precision + Recall}}.$$

## Results and discussions

We selected the Elliptic Bitcoin dataset^15^ which contains 157205 unlabeled nodes. It is not classified as licit or illicit class. Our goal was to find the best fit for our dataset so that we could identify the illicit transactions from the unlabeled nodes. We did GCN, Logistic Regression, Random Forest classifier and LSTM networks. We have created the GCN model using Adam as optimizer and loss as binary cross-entropy. We have set the learning rate to be 0.1. For LSTM, we have used Adam as optimizer and loss as Mean Squared Error. We set the number of epochs as five hundred for both. We have split the data into 70-30 training and testing for all models. A comparison between different models of the Elliptic Bitcoin data set is shown in Table 3. We get the following result.

**Table 3:** Comparison between models on Elliptic Bitcoin Dataset.

	ACCURACY	ROC-AUC	RMSE
GCN	0.9856	0.9444	0.1123
LR	0.9289	0.7907	0.2664
RF	0.9783	0.9401	0.1080
SVM	0.9681	0.7909	0.1784
LSTM	0.9752	0.8084	0.1200

**Fig. 13:**
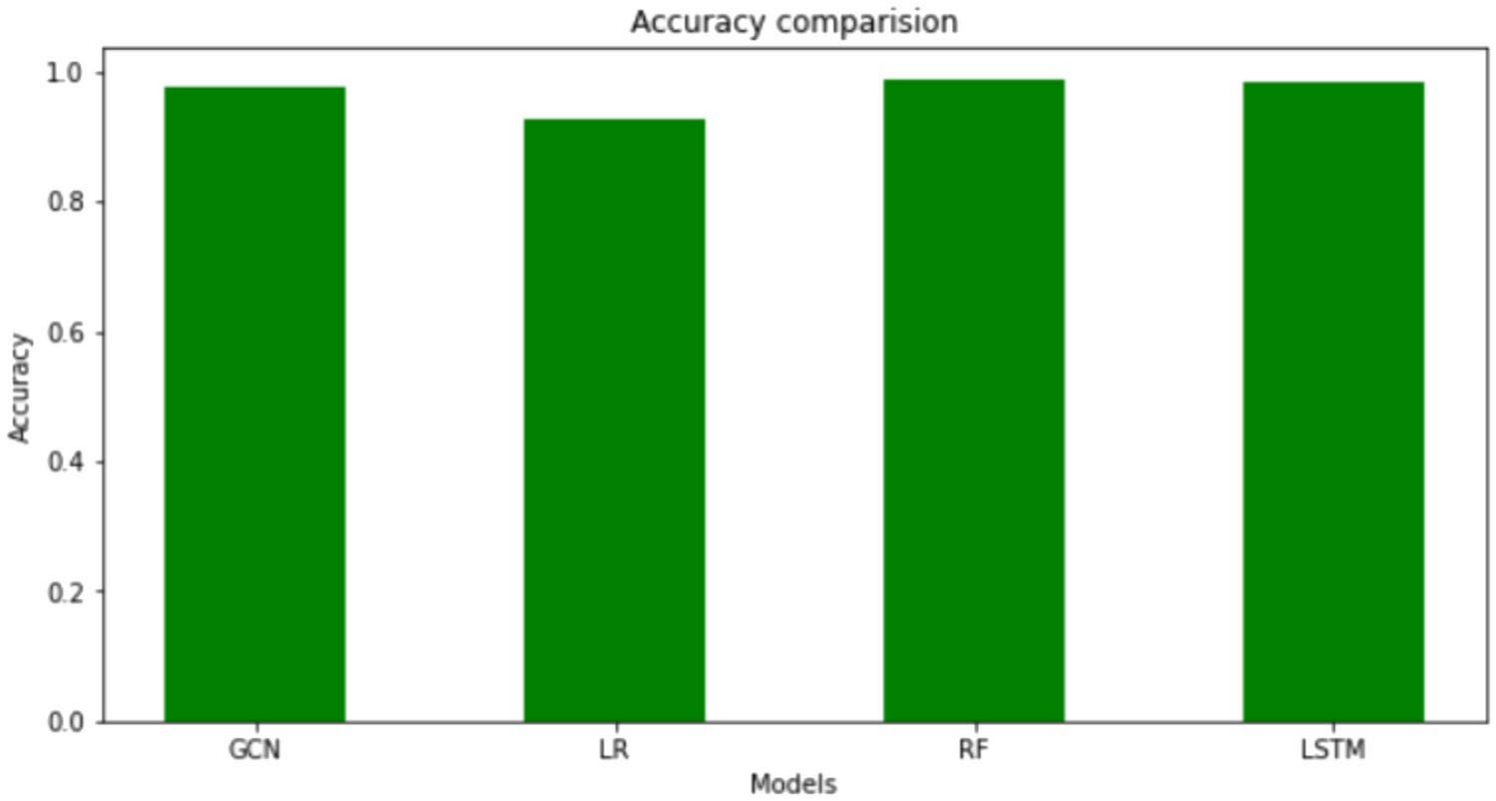
Accuracy comparison.

**Fig. 14:**
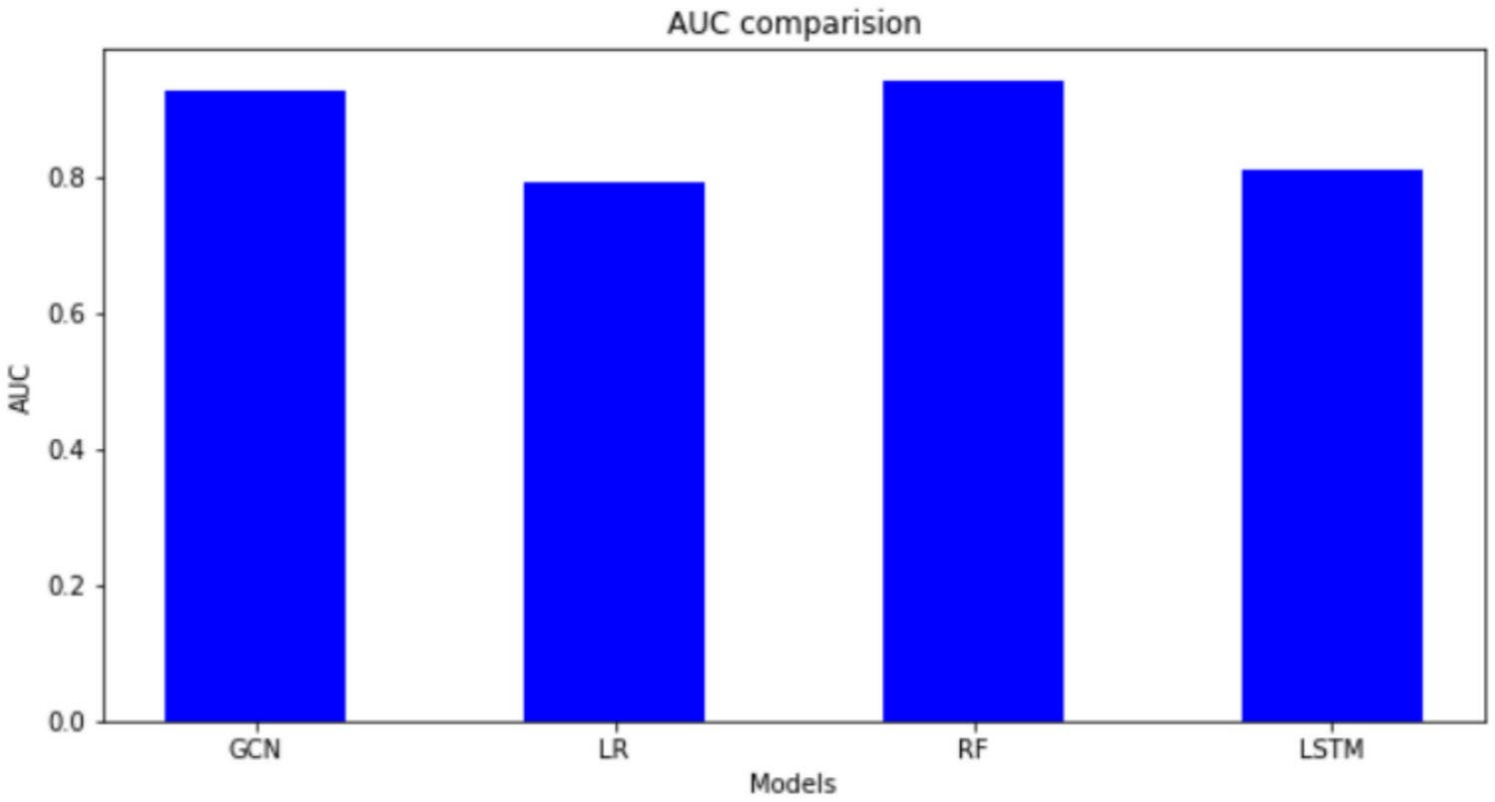
AUC comparison.

We can see that the GCN is best suited for our dataset though Random Forest follows very closely.

**Fig. 15:**
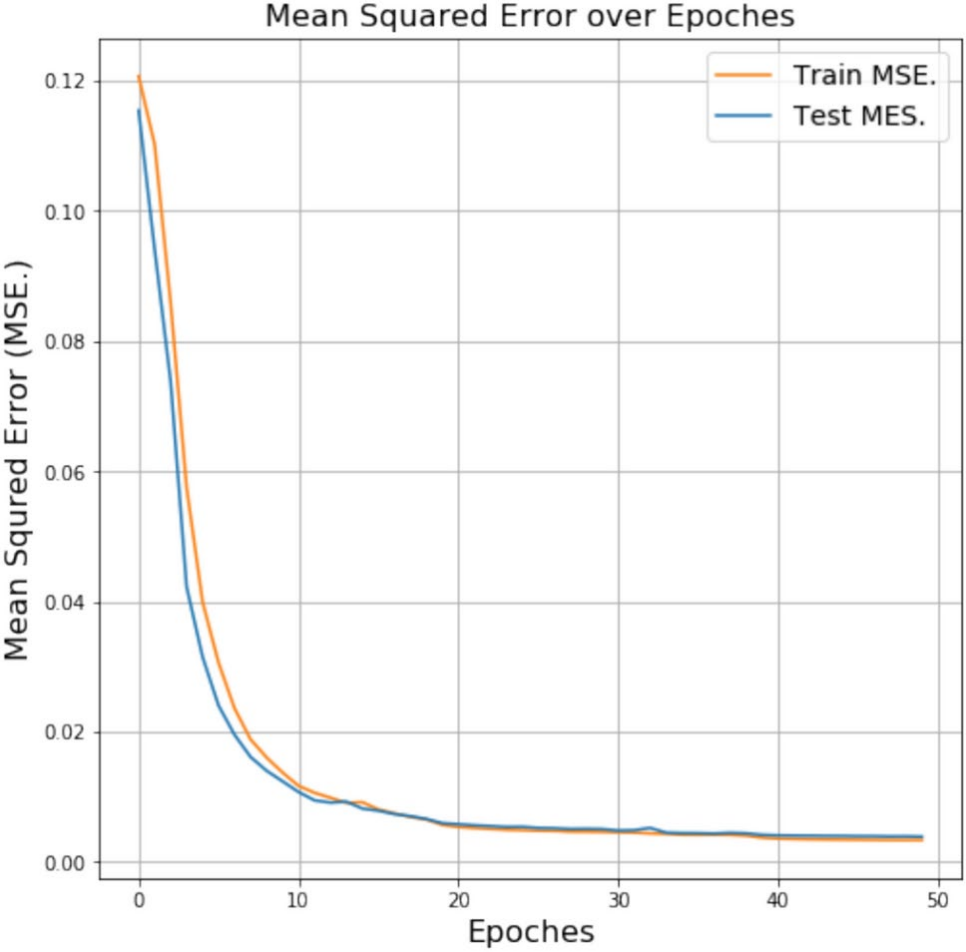
MSE for different Epoches.

**Fig. 16:**
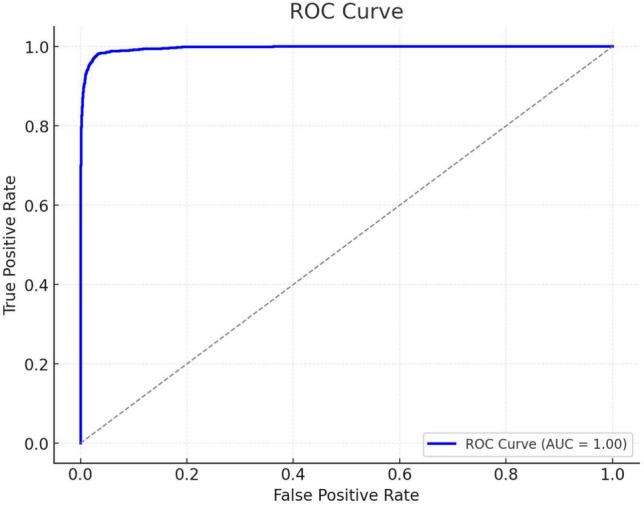
ROC curve.

The metrics we selected were accuracy, area under the receiver operator characteristic curve (AUC) score, and root mean square error (RMSE). Figure 13 shows the accuracy values of the different models. The AUC comparison is shown in Fig. 14. GCN has the highest accuracy and AUC score and the lowest RMSE score, making it a better algorithm. It is closely followed by LSTM in terms of accuracy and AUC score. The model that performed poorly when compared is Logistic Regression, though it is not as bad by itself. Compared with the other four algorithms except GCN in terms of the RMSE score, again RF has the better score. LR and SVM performed similarly in terms of AUC score, although SVM is better otherwise. Our proposed GCN model has the lowest score in every aspect except accuracy, though it is also a good algorithm on its own. We apply Mean Square Error (MSE) method for training and testing. Figure 15 shows MSE for different Epoches. Using this, we analyze the accuracy and loss of cross entropy. The ROC curve (Receiver Operating Characteristic curve) is a graphical representation of a binary classifier. This graph shows the relationship between the true positive and false positive rates for different values. We obtained the ROC graph for our data set and it is shown in Fig. 16. Also, Fig. 16 shows that the ACO is perfect (that is, the value of AOC is one).

### Comparison with other DL models

Pranav Nerurkar^14^ proposed a graph-based deep learning model to identify illegal transactions on bitcoin. In this session, we have shown different dep learning models and their results are discussed. The results are shown in Table 4.

**Table 4:** Comparison between DL models.

DL Models	Precision	Recall	F1 score (%)
VEG-16	0.9462	0.7438	75.45
ResNet	0.9543	0.7891	77.83
DenseNet-I	0.9589	0.8245	82.59
DenseNet-121	0.9611	0.8537	87.91
ResNet+DenseNet	0.9617	0.8794	88.63
DenseNet-II	0.9723	0.8962	89.54
CNN	0.9785	0.9126	91.23
GCN	0.9856	0.9381	92.87

The F1 score, calculated as the harmonic mean of precision and recall, reinforces the trends observed in the previous metric results. Nevertheless, the proposed GCN model shows the highest F1 score of 92. 87% compared to other DL models, which is shown in Fig. 19. We apply the paired *t* test for CNN and GCN to test their mean difference. The results of the paired *t* test indicated that there is a nonsignificant medium difference between CNN (mean is 31 and standard deviation is 52.1) and GCN (mean is 31.6 and standard deviation is 53.1) with *t* value one and the *p* value is 0.411.

**Fig. 17:**
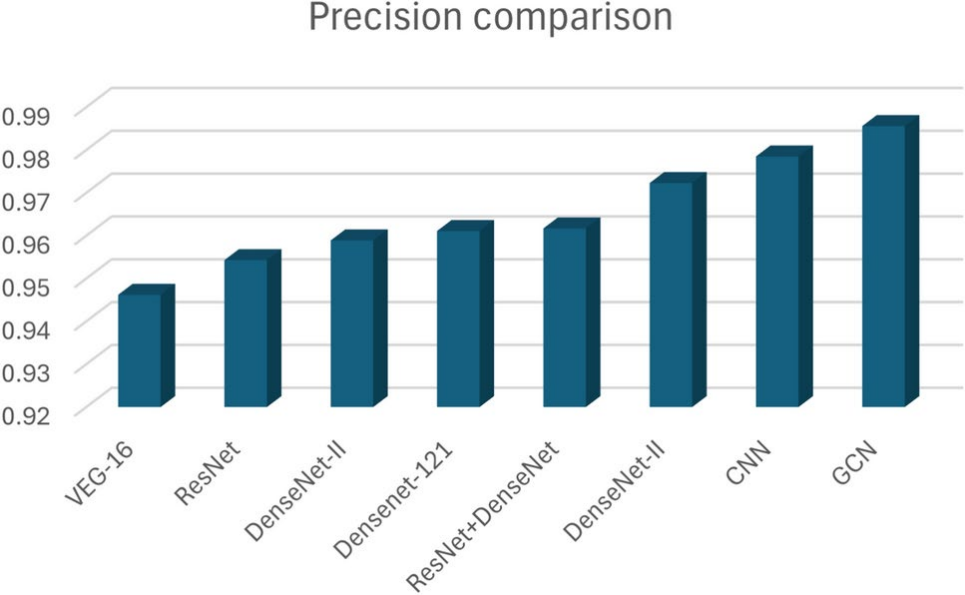
Precision comparison of Deep Learning models.

**Fig. 18:**
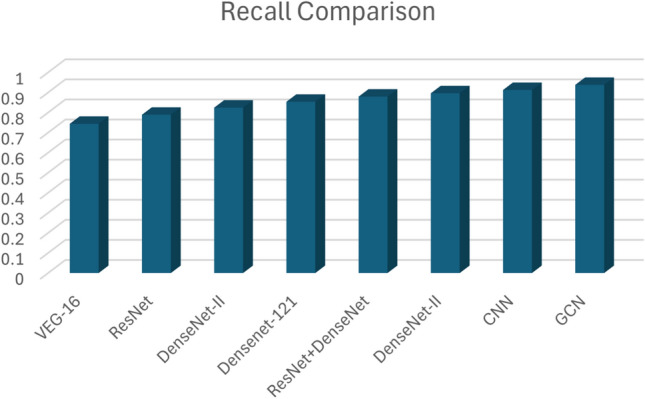
Recall comparisons of Deep Learning models.

**Fig. 19:**
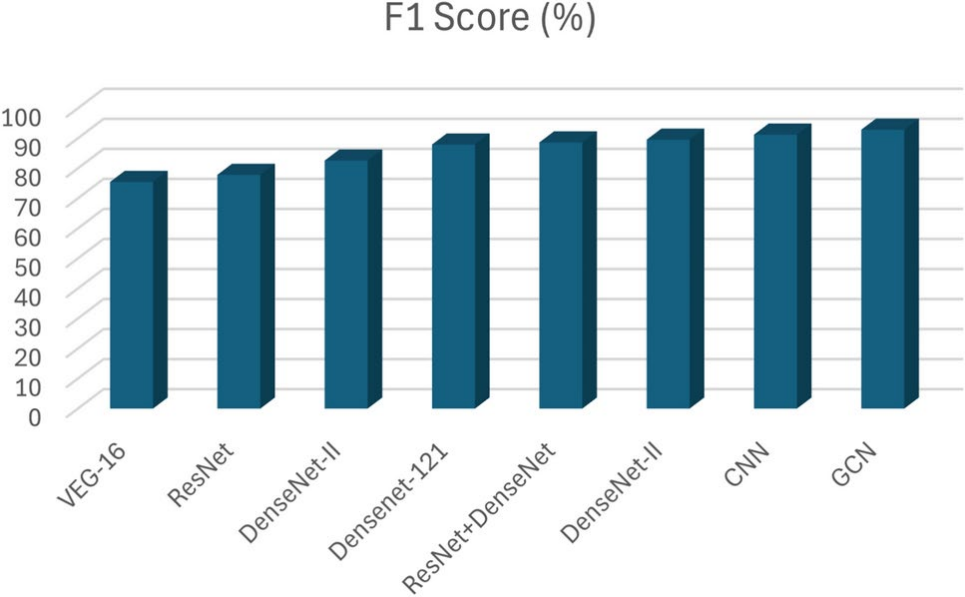
F1 score comparisons of Deep Learning models.

## Conclusions

Large group of people are affected by money laundering issues. It affects the business fields. Finding an efficient algorithm to detect the illicit transaction is an open problem. Many governments and private agencies are trying to find a solution to this cryptocurrency issue. Graph theory-based deep learning models are powerful for segmentation. It gives good accuracy and other parameters. In this paper, we proposed a Graph Convolution Network (GCN) to detect illicit transactions in the purchase and sale of cryptocurrency. We compare our GCN with other machine learning and deep learning algorithms. GCN gives better accuracy and other parameters. In addition, our GCN model shows a better performance than the models proposed in^14^.

There are several open problems in this research area. For example, ransomware detection in cryptocurrency transactions using deep leaning algorithms is an important problem for feature studies. In addition, identifying key players on the bitcoin network is an open problem.

## Data Availability

The datasets used and/or analyzed during the current study are available from the corresponding author on reasonable request.

## References

[1] Zhou, Y., Luo, X. & Zhou, M. C. Cryptocurrency transaction network embedding from static and dynamic perspectives: An overview. *IEEE/CAA J. Autom. Sin.* **10**(5), 1105–1121 (2023).

[2] Eyal, I. The miner’s dilemma. In *2015 IEEE Symposium on Security and Privacy*, 89–103 (2015).

[3] Eyal, I. & Sirer, E. G. Majority is not enough: Bitcoin mining is vulnerable. *Commun. ACM* **61**(7), 95–102 (2018).

[4] Kiayias, A., Koutsoupias, E., Kyropoulou, M. & Tselekounis, Y. Blockchain mining games. In *Proceedings of the 2016 ACM Conference on Economics and Computation, EC’16*, 365–382 (ACM, New York, NY, USA, 2016).

[5] Nakamoto, S. Bitcoin: A peer-to-peer electronic cash system. White Paper (2008). http://bitcoin.org/bitcoin.pdf.

[6] Weber, M. et al. Anti-money laundering in bitcoin: experimenting with graph convolutional networks for financial forensics. arXiv preprint arXiv:1908.02591 (2019).

[7] Bhaskar, N. D. & Chuen, D. L. E. E. K. Bitcoin exchanges. In *Handbook of Digital Currency*, 537–551 (Academic Press, 2024).

[8] Dhanya, N. M. An empirical evaluation of bitcoin price prediction using time series analysis and roll over. In *Inventive Communication and Computational Technologies*, 327–339 (Springer, Singapore, 2021).

[9] Badertscher, C., Maurer, U., Tschudi, D. & Zikas, V. Bitcoin as a transaction ledger: A composable treatment. *J. Cryptol.* **37**(2), 18 (2024).

[10] Kumar, P., Dhanush, G. A., Srivatsa, D., Nithin, A. & Sahisnu, S. A buyer and seller’s protocol via utilization of smart contracts using blockchain technology. In *International Conference on Advanced Informatics for Computing Research*, 464–474 (Springer, Singapore, 2019).

[11] Chen, Z. et al. Machine learning techniques for anti-money laundering (AML) solutions in suspicious transaction detection: A review. *Knowl. Inf. Syst.* **57**(2), 245–285 (2018).

[12] Grobys, K., Dufitinema, J., Sapkota, N. & Kolari, J. W. What’s the expected loss when Bitcoin is under cyberattack? A fractal process analysis. *J. Int. Financ. Mark. Inst. Money* **77**, 101534 (2022).

[13] Vlahavas, G., Karasavvas, K. & Vakali, A. Unsupervised clustering of bitcoin transactions. *Financ. Innov.* **10**(1), 25 (2024).

[14] Nerurkar, P. Illegal activity detection on bitcoin transaction using deep learning. *Soft. Comput.* **27**(9), 5503–5520 (2023).

[15] Elliptic, www.elliptic.co

[16] Weber, M. et al. *Anchorage* (AK, USA) (2019).

[17] Elmougy, Y., & Ling L. Demystifying fraudulent transactions and illicit nodes in the bitcoin network for financial forensics. In *Proceedings of the 29th ACM SIGKDD Conference on Knowledge Discovery and Data Mining*, 3979-3990. (2023).

[18] Maalouf, M. Logistic regression in data analysis: An overview. *Int. J. Data Anal. Tech. Strateg.* **3**(3), 281–299 (2011).

[19] Agresti, A. *An Introduction to Categorical Data Analysis* (John Wiley & Sons, Hoboken, 2018).

[20] Hastie, T., Tibshirani, R., Friedman, J. H. & Friedman, J. H. *The Elements of Statistical Learning: Data Mining, Inference, and Prediction* (Springer, New York, 2009).

[21] Hilbe, J. M. *Logistic Regression Models* (Chapman and hall/CRC, Boca Raton, 2009).

[22] Kim, H.-C., Pang, S., Je, H.-M., Kim, D. & Bang, S. Y. Constructing support vector machine ensemble. *Pattern Recognit.* **36**(12), 2757–2767 (2003).

[23] Suthaharan, S. Support vector machine. In *Machine Learning Models and Algorithms for Big Data Classification*, 207–235 (Springer, Boston, 2016).

[24] Backstrom, L. & Leskovec, J. Supervised random walks: Predicting and recommending links in social networks. In *Proceedings of the Fourth ACM International Conference on Web Search and Data Mining*, 635–644 (2011).

[25] Akoglu, L., Tong, H. & Koutra, D. Graph based anomaly detection and description a survey. *Data Min. Knowl. Disc.* **3**(29), 626–688 (2015).

[26] Zhang, S., Zhou, D., Yildirim, M. Y., Alcorn, S., He, J., Davulcu, H., & Tong H. Hidden: Hierarchical dense subgraph detection with application to financial fraud detection. In *Proceedings of the 2017 SIAM International Conference on Data Mining*, 570–578 (Society for Industrial and Applied Mathematics, 2017).

[27] Li, Y., Rose, Y., Shahabi, C. & Liu, Y. Diffusion Convolutional Recurrent Neural Network Data-Driven Traffic Forecasting International Conference on Learning. Representations (ICLR’18), 1–16 (2018).

[28] Monti, F. et al. Geometric deep learning on graphs and manifolds using mixture model CNNs. In *Proceedings of the IEEE Conference on Computer Vision and Pattern Recognition*, 5115–5124 (2017).

[29] Bruna, J., Zaremba, W., Szlam, A. & LeCun, Y. Spectral networks and locally connected networks on graphs. arXiv preprint arXiv:1312.6203 (2013).

[30] Li, Y., Tarlow, D., Brockschmidt, M. & Zemel, R. Gated graph sequence neural networks. arXiv preprint arXiv:1511.05493 (2015).

[31] Gilmer, J., Schoenholz, S. S., Riley, P. F., Vinyals, O. & Dahl, G. E. Neural message passing for quantum chemistry. In *International Conference on Machine Learning*, 1263–1272 (PMLR, 2017).

[32] Hamilton, W., Ying, Z. & Leskovec, J. Inductive representation learning on large graphs. In *Advances in Neural Information Processing Systems*, Vol. 30 (2017).

[33] Chanchal, M., Malathi, P. & Kumar, G. A comprehensive survey on Neural Network based Image Data hiding Scheme. In *2020 Fourth International Conference on I-SMAC (IoT in Social, Mobile, Analytics and Cloud)(I-SMAC)*, 1245–1249 (IEEE, 2020).

[34] Aarthi, R. & Harini, S. A survey of deep convolutional neural network applications in image processing. *Int. J. Pure Appl. Math* **118**, 185–190 (2018).

[35] Defferrard, M., Bresson, X. & Van der Gheynst, P. Convolutional neural networks on graphs with fast localized spectral filtering. In *Proc. NIPS*, 3844–3852 (2016).

[36] Wu, Z. et al. A comprehensive survey on graph neural networks. *IEEE Trans. Neural Netw. Learn. Syst.* **32**(1), 4–24 (2020). 10.1109/TNNLS.2020.2978386 32217482

